# Covalent Bonds *versus* van der Waals Forces: A Picture in Thermal Conduction of Organic Materials

**DOI:** 10.1021/jacs.4c11849

**Published:** 2024-10-24

**Authors:** Ryosuke Takehara, Tomoya Fukui, Taketo Tano, Meguya Ryu, Suguru Kitani, Hitoshi Kawaji, Junko Morikawa, Takanori Fukushima

**Affiliations:** 1 Laboratory for Chemistry and Life Science, Institute of Innovative Research, Tokyo Institute of Technology, 4259 Nagatsuta, Midori-ku, Yokohama 226-8501, Japan; 2 Department of Chemical Science and Engineering, School of Materials and Chemical Technology, Tokyo Institute of Technology, 4259 Nagatsuta, Midori-ku, Yokohama 226-8501, Japan; 3 Research Center for Autonomous Systems Materialogy (ASMat), Institute of Innovative Research, Tokyo Institute of Technology, 4259 Nagatsuta, Midori-ku, Yokohama 226-8501, Japan; 4 National Methodology Institute of Japan (NMIJ), Advanced Industrial Science and Technology (AIST), Tsukuba Central 3, 1-1-1 Umezono, Tsukuba 305-8563, Japan; 5 Materials and Structures Laboratory, Tokyo Institute of Technology, 4259 Nagatsuta-cho, Midori-ku, Yokohama 226-8501, Japan; 6 Department of Materials Science and Engineering, School of Materials and Chemical Technology, Tokyo Institute of Technology, 2-12-1 Ookayama, Meguro-ku, Tokyo 152-8550, Japan

## Abstract

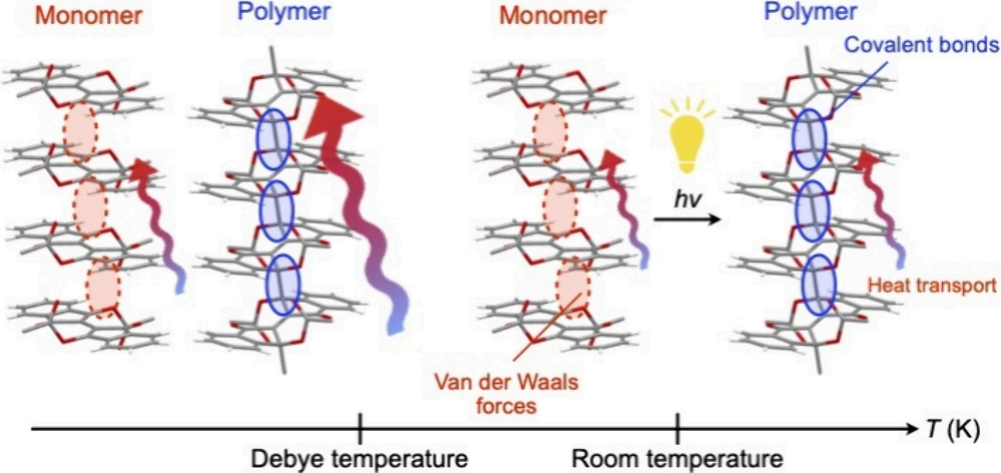

We present a direct comparison of the heat transport properties between the state in which the constituent molecules are assembled by intermolecular forces and the one in which they are covalently bonded, in a molecular system with identical constituent elements and masses, as well as a nearly identical structure and density. This comparison leading to an essential understanding of thermal conduction in organic materials is made possible by the unique compound found by Wudl et al., which exhibits a single-crystal-to-single-crystal topochemical polymerization with a yield of >99%, in combination with microtemperature wave analysis (*μ*TWA), which allows accurate measurements of the thermal diffusivity of small single crystals. At room temperature, the thermal conductivity of monomer and polymer single crystals is not significantly different. For both crystals, the thermal conductivity increases monotonically with decreasing temperature. However, below the Debye temperature, the thermal conductivity of the polymer single crystal increases exponentially, giving much larger values than those of the monomer single crystal. Based on physical quantities related to the behavior of phonons, derived from the specific heat analysis, we discuss the differences in heat transport properties in the two states and provide guidelines for achieving high thermal conductivity in organic materials.

## Introduction

Phonons are responsible for heat transport in materials where free electrons are absent.^1^ Therefore, unlike inorganic and carbon materials in which atoms are infinitely linked by ionic and covalent bonds, in organic materials, which are substances where the constituent molecules are assembled through weak intermolecular forces, heat transport by phonons does not efficiently occur. Furthermore, since molecules themselves are substances, in which multiple elements are linked in various bonding modes, and have complex inherent vibrational modes, the heat energy stored as intramolecular vibrations is not necessarily involved in heat transport.^2^ Reflecting these facts, it is commonly believed that organic materials do not intrinsically exhibit high thermal conduction.^3−9^

**Figure 1:**
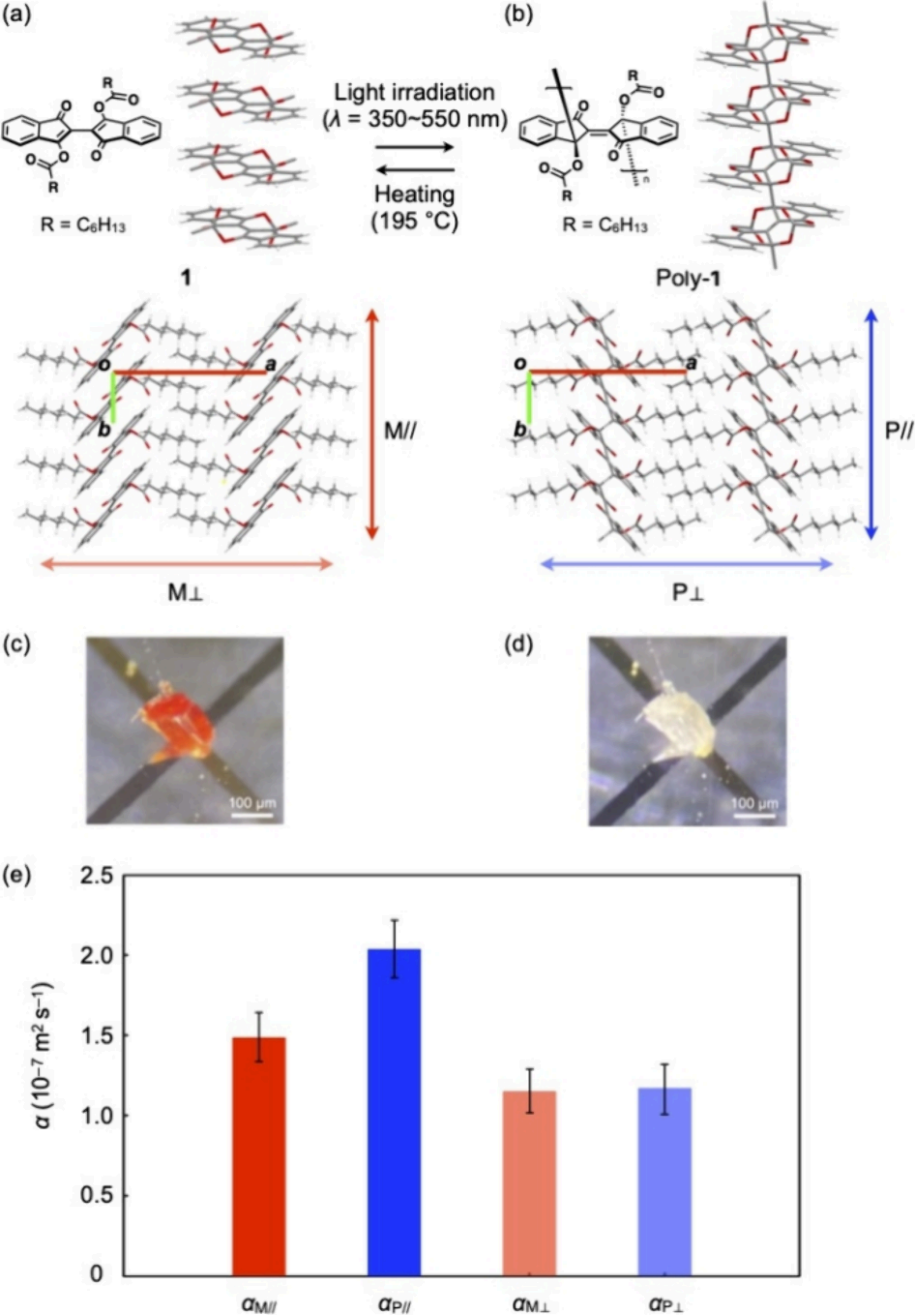
Schematic chemical structures and the X-ray crystal structure of (a) monomer **1** and (b) poly-**1**. The X-ray structures, which have been obtained from our own single-crystal X-ray analysis, are shown (see the Supporting Information). For the substituents (R = C_6_H_13_), only the methylene moiety attached to the carbonyl group is shown for clarity. The bond distances between the carbon atoms at the 3,3′-positions in **1** and poly-**1** are 3.306 and 1.598 Å, respectively, which agree with those reported by Wudl et al. The direction, in which the thermal diffusivity (*α*) was measured, is indicated by colored arrows. Photographs of single crystals of (c) **1** and (d) poly-**1** on a Au–Ni sensor in the experimental setup for *μ*TWA measurements. (e) Thermal diffusivity values of *α*_M//_ (red), *α*_P//_ (blue), *α*_M⊥_ (light red), and *α*_P⊥_ (light blue) at 300 K.

Now, what would be the thermal conductivity characteristics if molecules were infinitely linked by covalent bonds? To examine this idea, it is necessary to find an organic material that meets three requirements: (1) it must be of sufficient size to enable experimental thermal conductivity measurements; (2) it must have two structurally characterizable states, one covalently bonded and one not, to allow for comparative verification; (3) other factors related to the constituent molecules, including mass, elements present, and assembly structure, must be almost identical in these two states. To the best of our knowledge, only one system that satisfies these strict requirements has been reported thus far, namely, [2,2′-bi-1*H*-indene]-3,3′-dihydroxy-1,1′-heptanoate (**1**, Figure 1a), discovered by Wudl et al. in 2014.^10^ This molecule undergoes quantitative (>99%) single-crystal-to-single-crystal topochemical polymerization into poly-**1** (Figure 1b) upon exposure to visible light (*λ* = 350–550 nm) and depolymerization upon heating at 195 °C, to recover monomer **1** while maintaining its single crystalline form.

Here, we describe the heat transport properties and their temperature dependence below room temperature for single crystals of monomer **1** and poly-**1**. We obtained the thermal diffusivity of **1** and poly-**1** at each temperature using a microtemperature wave analysis (*μ*TWA) technique,^11,12^ which allows for the accurate evaluation of thermal diffusivity and its anisotropy even for micrometer-sized small single crystals.^13^ Using the experimentally obtained thermal diffusivity, specific heat, and density, the thermal conductivity of **1** and poly-**1** was determined. Interestingly, while the difference in thermal conductivity between **1** and poly-**1** is small at high temperatures, below their Debye temperatures, a significant increase in thermal conductivity was observed only for poly-**1**. The origin of this behavior is discussed.

## Results and Discussion

Monomer **1** and its single crystals were prepared by following the reported procedures.^10^ When orange-colored single crystals of **1** were exposed to a white LED light (OLYMPUS, SZ-LW61), topochemical polymerization took place to give colorless single crystals of poly-**1** (Figure 1c,d). The quantitative conversion of **1** into poly-**1** was confirmed by diffuse reflectance spectroscopy as well as extraction of the polymer crystals using dichloromethane as reported previously.^10^

By means of *μ*TWA, we evaluated thermal diffusivities for single crystals of **1** and poly-**1** in the directions parallel (*α*_M//_ and *α*_P//_) and perpendicular (*α*_M⊥_ and *α*_P⊥_) to the polymer chains (i.e., the molecular stacking direction for **1**). The values of *α*_M//_, *α*_P//_, *α*_M⊥_, and *α*_P⊥_ at 300 K were determined to be 1.47 ± 0.14 × 10^–7^, 2.01 ± 0.18 × 10^–7^, 1.16 ± 0.14 × 10^–7^, and 1.17 ± 0.16 × 10^–7^ m^2^ s^–1^, respectively (Figure 1e). We originally predicted that the poly-**1** crystal in which the monomer units are covalently bonded should have a much higher thermal diffusivity than the monomer **1** crystal in which only intermolecular forces operate. However, no significant difference in thermal diffusivity was observed before and after polymerization or with changes in the polymer chain direction, although the value of *α*_P//_ is higher than in other cases. Thus, covalent bonding between the monomer units leads to some improvement in the heat transport properties in the crystalline state, but not to any great extent at room temperature, giving a thermal diffusivity change of *α*_P//_/*α*_M//_ = 1.4.

**Figure 2:**
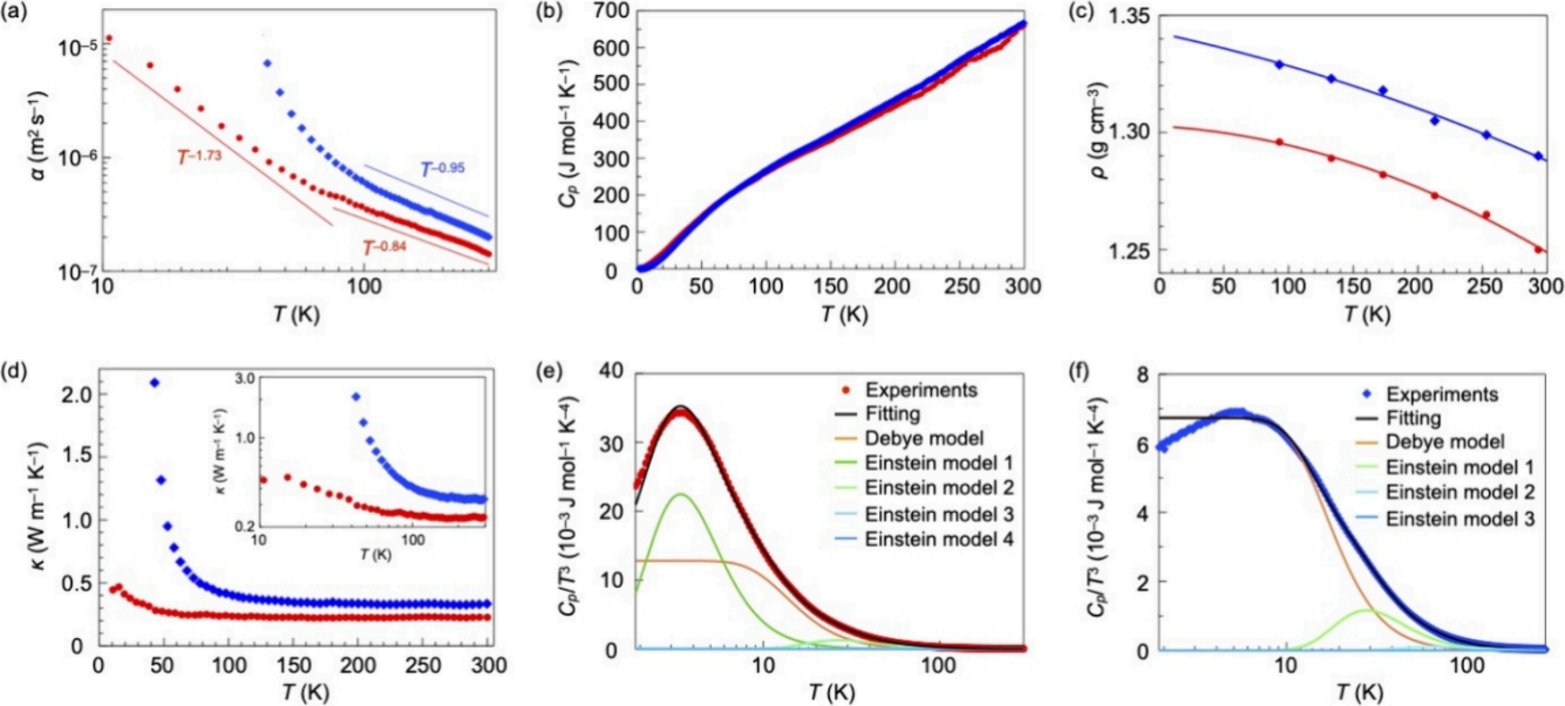
(a) Temperature dependence of the thermal diffusivity of **1** (red circle, *α*_M//_) and poly-**1** (blue diamonds, *α*_P//_) in the direction parallel to the polymer chains. They are plotted as ln *α*_M//_ and ln *α*_P//_ against ln *T*. Hereafter, red circles and blue diamonds represent the data for **1** and poly-**1**, respectively. (b) Temperature dependence of the specific heat (*C*_*p*_) of **1** and poly-**1** measured using the relaxation method. (c) Temperature dependence of the density of **1** and poly-**1** obtained by single-crystal X-ray crystallography, along with their extrapolation curves. (d) Temperature dependence of the thermal conductivity of **1** (*κ*_M//_) and poly-**1** (*κ*_P//_); log–log plots are shown in the inset. Plots of experimentally obtained *C*_*p*_/*T*^3^ values and those calculated using the Debye and Einstein specific heat models for (e) **1** and (f) poly-**1**, against *T*.

Notably, when the temperature is decreased, a large difference in the thermal diffusivity before and after polymerization is observed. As shown in Figure 2a, the values of *α*_M//_ (red) and *α*_P//_ (blue) increase monotonically in proportion to *T*^–0.84^ (at 80–300 K) and *T*^–0.95^ (at 100–300 K), respectively, with decreasing temperature. This behavior is due to an increase in the scattering time of thermal carriers, and both exponents are close to −1, indicating that phonons are scattered by the Umklapp process.^14−16^ Note that the change in thermal diffusivity of monomer **1** and poly-**1** with temperature becomes abrupt below a certain point. While the temperature dependence of *α*_M//_ below 80 K appears to be roughly in proportion to *T*^–1.73^, that of *α*_P//_ below 100 K does not follow a power law. Obviously, the influence of the covalent bonding between the monomers on the behavior of phonon scattering is pronounced in the low temperature region.

To investigate the behavior of thermal conductivity, we also measured the temperature dependence of specific heat at constant pressure (*C*_*p*_) and density (*ρ*) of monomer **1** (red) and poly-**1** (blue) (Figure 2b,c). The *C*_*p*_ values are almost identical to each other, meaning that the monomer and polymer in their crystalline states have the same number of degrees of freedom, with no significant difference in the intra- and intermolecular energies corresponding to each degree of freedom. The *ρ* values of **1** and poly-**1** at selected temperatures were determined by single-crystal X-ray crystallography. As expected, after topochemical polymerization, the density increases.

Using the experimentally obtained values of thermal diffusivity (*α*_M//_ and *α*_P//_), *C*_*p*_, and *ρ*, we obtained the temperature dependence of thermal conductivity (*κ* = *αC*_*p*_*ρ*) in the corresponding directions (*κ*_M//_ and *κ*_P//_). As shown in Figure 2d, at 294 K, *κ*_M//_ (red) and *κ*_P//_ (blue) are 0.24 and 0.33 W m^–1^ K^–1^, respectively, giving a ratio of *κ*_P//_/*κ*_M//_ = 1.4. In the temperature range of 100–300 K, the values of *κ*_M//_ and *κ*_P//_ are not significantly different. However, below 100 K, a remarkable change in *κ*_M//_ and *κ*_P//_ was observed. For example, at 43 K, the values of *κ*_M//_ and *κ*_P//_ were determined to be 0.29 and 2.10 W m^–1^ K^–1^, respectively. Thus, the thermal conductivity of poly-**1** becomes 7.2 times larger compared with that at 294 K. Importantly, the temperature, at which *κ*_P//_/*κ*_M//_ begins to abruptly increase, roughly corresponds to the Debye temperatures (*θ*_D_) of **1** (67.2 K) and poly-**1** (83.3 K), obtained from the analysis of the temperature dependence of their *C*_*p*_ values as described below.

The Debye temperature is a physical quantity unique to a material, and by determining the Debye temperatures for monomer **1** and poly-**1**, their phonon group velocity and mean free paths can be evaluated. For molecular crystals, the specific heat at constant volume (*C*_*v*_) can be represented by both Debye and Einstein specific heat models.^17^ The former describes *C*_*v*_, originating from the three translational and three rotational degrees of freedom when a molecule is considered as a rigid body,^18^ and is expressed as
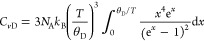
where *C*_*v*D_, *N*_A_, *k*_B_, and *θ*_D_ are the Debye specific heat at constant volume, the Avogadro constant, the Boltzmann constant, and the Debye temperature, respectively.^19^ The latter describes *C*_*v*_ originating from intramolecular degrees of freedom. The Einstein specific heat model is expressed as
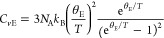
where *C*_*v*E_ and *θ*_E_ are the Einstein specific heat at constant volume and the Einstein temperature, respectively.^20^

Using these two models, we obtained the Debye temperatures of **1** and poly-**1**, both of which consist of 72 atoms, and thus, there is a total of 216 degrees of freedom. For simplicity, we treat each of the three translational (trans) and three rotational (rot) degrees of freedom with one Debye specific heat model (*C*_*v*D_trans_ and *C*_*v*D_rot_), and four Einstein specific heat models (*C*_*v*E*i*_, *C*_*v*E*j*_, *C*_*v*E*k*_, and *C*_*v*E*l*_; *i*, *j*, *k*, and *l* = the number of intramolecular vibrational modes) are applied to the remaining 210 intramolecular degrees of freedom.^2^ Accordingly, the total specific heat (*C*_*v*_) can be expressed as
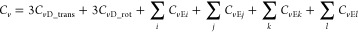
where *i* + *j* + *k* + *l* = 210.

Since *C*_*v*_ is difficult to obtain experimentally and the difference between *C*_*p*_ and *C*_*v*_ in a real system is considered to be small,^2^ we analyzed the experimentally obtained *C*_*p*_ curve (Figure 2e,f) using the three equations, where *θ*_D_, *θ*_E_, *i*, *j*, *k*, and *l* were used as parameters. The best fit was obtained when the values of *C*_*v*D_trans_ and *C*_*v*D_rot_ were equal, giving Debye temperatures (*θ*_D_) of 67.2 and 83.3 K for monomer **1** and poly-**1**, respectively. These temperatures roughly coincide with those at which the thermal diffusivity changes abruptly (Figure 2a). Regarding the four Einstein temperatures (*θ*_E_), the *C*_*p*_ curve of **1** (Figure 2e) was best fitted with 16.7, 132.3, 341.4, and 1378.4 K. On the other hand, the *C*_*p*_ curve of poly-**1** (Figure 2f) was better reproduced when fitting with three parameters (*i*, *j*, and *k*) than with four (*i*, *j*, *k*, and *l*), and thus, the three values of *θ*_E_ were successfully determined to be 141.0, 352.5, and 1370.0 K. The peak observed around 30 K for **1** (Figure 2e) is due to a vibrational mode with an energy of 16.7 K, while the corresponding peak is absent for poly-**1** (Figure 2f). This suggests that the covalent bonding between the monomeric **1** induces a shift of the intramolecular vibrational modes to a higher energy region.

Assuming isotropic phonon dispersions, we estimated phonon group velocity (*v*_ph_) using the equation
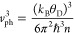
where *k*_*B*_ is the Boltzmann constant, ℏ is the Dirac constant, and *n* is the number density of molecules. Based on this equation, the values of *v*_ph_ were calculated to be 1970 and 2420 m s^–1^ for **1** and poly-**1**, respectively. The ratio of *v*_ph_ before and after polymerization is 1.2, which is in good agreement with the experimental observation (*κ*_P//_/*κ*_M//_ = 1.4 at 294 K, Figure 2d).^21^ Thus, the large difference in thermal conductivity between **1** and poly-**1** in the lower temperature region (<100 K) cannot be explained in terms of phonon group velocity. We considered that this difference is due to changes in the mean free path (*l*) and scattering time (*τ*) of phonons, rather than the phonon group velocity.

Using the equations^22^
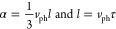
the values of *l* and *τ* for **1** and poly-**1** at 300 and 43 K were calculated (Table 1). The calculated mean free path and scattering time of phonons are similar before and after polymerization around room temperature. It is noteworthy, however, that the difference in these values between **1** and poly-**1** increases with decreasing temperature. At 43 K, poly-**1** (83.8 Å) has a 5.8 times larger *l* value than **1** (14.4 Å), and the value of *τ* for poly-**1** (34.6 × 10^–13^ s) is 4.7 times larger compared with **1** (7.31 × 10^–13^ s). Clearly, the difference in heat transport properties depending on whether the monomer units are assembled by intermolecular forces or linked by covalent bonds becomes apparent only in the region below the Debye temperature (*θ*_D_ = 67.2 and 83.3 K for monomer **1** and poly-**1**, respectively).

**Table 1:** Values of *v*_ph_, *l* (at 300 and 43 K), and τ (at 300 and 43 K) for Monomer**1** and Poly-**1**

		300 K		43 K	
	*v*_ph_ (m s^–1^)	*l* (Å)	*τ* (s)	*l* (Å)	*τ* (s)
**1**	1970	2.23	1.13 × 10^–13^	14.4	7.31 × 10^–13^
poly-**1**	2420	2.49	1.03 × 10^–13^	83.8	34.6 × 10^–13^

When the temperature drops below *θ*_D_, phonon excitation is suppressed, resulting in increases in both *l* and *τ*. It is well-known that at high temperatures, *l* and *τ* follow a power law with temperature, while below *θ*_D_, they behave in an exponential manner [exp(*cθ*_D_/*T*), *c* = constant].^15,23^ For thermal conduction to take on finite values, in other words, for thermal resistance to occur, phonons must undergo Umklapp scattering, in which crystal momentum including the reciprocal lattice vector must be conserved. When Umklapp scattering takes place below *θ*_D_, at least one phonon with energy as large as *θ*_D_ should be involved in the scattering process. Below *θ*_D_, the existence probability of such a phonon is proportional to exp(−*cθ*_D_/*T*). This means that the lower the temperature, the fewer the amount of such phonons present, leading to an increase in both *τ* and *l* (*l* = *v*_Ph_*τ*), which both increase exponentially with the function exp(*cθ*_D_/*T*). Due to this exponential behavior, a small change in *θ*_D_ results in drastic changes in *τ* and *l*. Looking at the experimental results (Figure 2a), while **1** with a lower *θ*_D_ (67.2 K) displays a gradual exponential curve, poly-**1** with a higher *θ*_D_ (83.3 K) shows pronounced exponential behavior. The Debye temperatures of substances comprehensively reflect their inherent structural parameters. In the present system, before and after topochemical polymerization, the types and number of elements are the same, the crystal structures are isomorphous, and the change in molecular structure is rather small. Therefore, the change in *θ*_D_ between **1** and poly-**1** is exclusively due to the formation of covalent bonds. This provides an increase in *θ*_D_, which in turn results in a significant improvement in the heat transport properties below *θ*_D_.

## Conclusions

Thanks to a molecule discovered by Wudl et al. that exhibits a single-crystal-to-single-crystal topochemical polymerization with a yield of >99% and the *μ*TWA method, which allows for accurate measurements of the thermal diffusivity of small single crystals, we have successfully shown for the first time the difference in thermal conduction between molecules assembled by intermolecular forces and those linked by covalent bonds. Covalent bonds and intermolecular forces are essential in organic materials, while heat is a ubiquitous energy. Thus, understanding of their relationship is important for the development of functional organic materials, especially those for thermal management. It is intuitively understandable that polymers have better heat transport properties than low-molecular-weight assemblies, but in reality, even with the ingenuity of polymer synthesis, attempts to improve their heat transport properties have often been unsuccessful. This work, which clearly demonstrates the importance of the Debye temperature, provides useful guidelines for the design of highly thermally conductive organic and polymeric materials.

## Supporting Information Available

Experimental details and methods including single-crystal X-ray analysis, specific heat measurements, and thermal diffusivity measurements using a microtemperature wave analysis (*μ*TWA) (PDF)
