# Supplementary material for: Covalent Bonds versus van der Waals Forces: A Picture in Thermal Conduction of Organic Materials
Source: J Am Chem Soc. 2024 Oct 24;146(44):30548–52. doi: 10.1021/jacs.4c11849 (PMC11544605; doi:10.1021/jacs.4c11849)
Supplement: Supplementary file 1 — ja4c11849_si_001.pdf [file ja4c11849_si_001.pdf]

Supporting Information

Covalent Bonds versus van der Waals Forces: A Picture in Thermal Conduction of Organic Materials

Ryosuke Takehara, Tomoya Fukui, Taketo Tano, Meguya Ryu, Suguru Kitani, Hitoshi Kawaji, Junko Morikawa,* and Takanori Fukushima*

*To whom correspondence should be addressed.
E-mail: fukusihma@res.titech.ac.jp (T.F.) and morikawa.j.aa@m.titech.ac.jp (J.M.)

Table of Contents

1. Materials **S2**
2. Methods **S2**
3. Supporting Figure **S5**
4. Supporting Tables **S6**
5. Supporting References **S7**

1. Materials

Unless otherwise stated, all commercial reagents were used as received. Single crystals of **1** and poly-**1** were prepared according to the previously reported procedures.[S1]

2. Methods

Single-crystal X-ray analysis. A single crystal sample of **1** or poly-**1** was coated with immersion oil (type B: Code 1248, Cargille Laboratories, Inc.) and mounted on a MicroMount (MiTeGen, LLC.). Diffraction data were collected at given temperatures under a cold nitrogen gas stream on a RIGAKU model XtaLAB Synergy-DW diffractometer system equipped with a HyPix-6000 detector, using Cu-$K\alpha$ radiation (λ = 1.54184 Å). Based on the X-ray crystallographic data (Table S1), the temperature-dependent volume change was evaluated from 93 to 293 K at intervals of 40 K. The density at each temperature was calculated from the molecular weight of **1** (MW = 514.62) or that of repeating unit of poly-**1** (MW = 514.62) and the number of molecules in the unit cell (Z = 2). The temperature dependence of the density below 93 K and above 293 K was extrapolated with the quadratic functions.[S2]

Crystal data for 1 at 93 K: red needle, monoclinic, $P2_1/c$, a = 13.9686(5) Å, b = 4.89373(17) Å, c = 19.6219(6) Å, β = 100.498(3)°, V = 1318.87(8) Å^3, Z = 2, density$_{calcd}$ = 1.296 g cm^{-3}, T = 93 K, Cu$K\alpha$ radiation, λ = 1.54184 Å, μ = 0.716 mm^{-1}, R_{int} = 0.0156, GOF = 1.045, R_1 = 0.0418 (I > 2σ(I)), wR_2 = 0.1114 (all data), $\Delta\rho_{max,min}$ = 0.361, –0.156 eÅ^3.

Crystal data for 1 at 133 K: red needle, monoclinic, $P2_1/c$, a = 14.0204(5) Å, b = 4.89521(19) Å, c = 19.6535(8) Å, β = 100.670(4)°, V = 1325.55(9) Å^3, Z = 2, density$_{calcd}$ = 1.289 g cm^{-3}, T = 133 K, Cu$K\alpha$ radiation, λ = 1.54184 Å, μ = 0.713 mm^{-1}, R_{int} = 0.0149, GOF = 1.035, R_1 = 0.0391 (I > 2σ(I)), wR_2 = 0.0993 (all data), $\Delta\rho_{max,min}$ = 0.337, –0.149 eÅ^3.

Crystal data for 1 at 173 K: red needle, monoclinic, $P2_1/c$, a = 14.0820(7) Å, b = 4.8982(2) Å, c = 19.6743(10) Å, β = 100.774(5)°, V = 1333.16(11) Å^3, Z = 2, density$_{calcd}$ = 1.282 g cm^{-3}, T = 173 K, Cu$K\alpha$ radiation, λ = 1.54184 Å, μ = 0.709 mm^{-1}, R_{int} = 0.0120, GOF = 1.051, R_1 = 0.0406 (I > 2σ(I)), wR_2 = 0.1099 (all data), $\Delta\rho_{max,min}$ = 0.336, –0.140 eÅ^3.

Crystal data for 1 at 213 K: red needle, monoclinic, $P2_1/c$, a = 14.1596(6) Å, b = 4.8989(2) Å, c = 19.7111(7) Å, β = 100.932(4)°, V = 1342.49(10) Å^3, Z = 2, density$_{calcd}$ = 1.273 g cm^{-3}, T = 213 K, Cu$K\alpha$ radiation, λ = 1.54184 Å, μ = 0.704 mm^{-1}, R_{int} = 0.0168, GOF = 1.057, R_1 = 0.0394 (I > 2σ(I)), wR_2 = 0.0997 (all data), $\Delta\rho_{max,min}$ = 0.260, –0.115 eÅ^3.

Crystal data for 1 at 253 K: red needle, monoclinic, $P2_1/c$, a = 14.2422(8) Å, b = 4.8999(3) Å, c = 19.7288(10) Å, β = 101.110(5)°, V = 1350.97(13) Å^3, Z = 2, density$_{calcd}$ = 1.265 g cm^{-3}, T = 253 K, Cu$K\alpha$ radiation, λ = 1.54184 Å, μ = 0.699 mm^{-1}, R_{int} = 0.0150, GOF = 1.053, R_1 = 0.0391 (I > 2σ(I)), wR_2 = 0.1083 (all data), $\Delta\rho_{max,min}$ = 0.230, –0.122 eÅ^3.

Crystal data for 1 at 293 K: red needle, monoclinic, $P2_1/c$, a = 14.3310(6) Å, b = 4.9168(2) Å, c

= 19.7877(8) Å, β = 101.299(4)°, V = 1367.27(11) Å^3, Z = 2, density$_{calcd}$ = 1.250 g cm^{-3}, T = 293 K, Cu$K\alpha$ radiation, λ = 1.54184 Å, μ = 0.691 mm^{-1}, R_{int} = 0.0194, GOF = 1.075, R_1 = 0.0385 ($I > 2\sigma(I)$), wR_2 = 0.1092 (all data), $\Delta\rho_{max,min}$ = 0.127, –0.123 eÅ^3.

Crystal data for poly-1 at 93 K: colorless needle, monoclinic, $P2_1/c$, a = 14.248(3) Å, b = 4.8545(7) Å, c = 19.207(3) Å, β = 104.594(17)°, V = 1285.6(4) Å^3, Z = 2, density$_{calcd}$ = 1.329 g cm^{-3}, T = 93 K, Cu$K\alpha$ radiation, λ = 1.54184 Å, μ = 0.735 mm^{-1}, R_{int} = 0.0372, GOF = 1.157, R_1 = 0.0694 ($I > 2\sigma(I)$), wR_2 = 0.1731 (all data), $\Delta\rho_{max,min}$ = 0.23, –0.26 eÅ^3.

Crystal data for poly-1 at 133 K: colorless needle, monoclinic, $P2_1/c$, a = 14.296(3) Å, b = 4.8576(7) Å, c = 19.240(3) Å, β = 104.852(17)°, V = 1291.5(4) Å^3, Z = 2, density$_{calcd}$ = 1.323 g cm^{-3}, T = 133 K, Cu$K\alpha$ radiation, λ = 1.54184 Å, μ = 0.731 mm^{-1}, R_{int} = 0.0396, GOF = 1.049, R_1 = 0.0593 ($I > 2\sigma(I)$), wR_2 = 0.1493 (all data), $\Delta\rho_{max,min}$ = 0.23, –0.26 eÅ^3.

Crystal data for poly-1 at 173 K: colorless needle, monoclinic, $P2_1/c$, a = 14.3762(13) Å, b = 4.8544(3) Å, c = 19.2469(12) Å, β = 105.148(8)°, V = 1296.52(17) Å^3, Z = 2, density$_{calcd}$ = 1.318 g cm^{-3}, T = 173 K, Cu$K\alpha$ radiation, λ = 1.54184 Å, μ = 0.729 mm^{-1}, R_{int} = 0.0330, GOF = 1.086, R_1 = 0.0563 ($I > 2\sigma(I)$), wR_2 = 0.1381 (all data), $\Delta\rho_{max,min}$ = 0.200, –0.227 eÅ^3.

Crystal data for poly-1 at 213 K: colorless needle, monoclinic, $P2_1/c$, a = 14.4786(6) Å, b = 4.85837(17) Å, c = 19.3038(7) Å, β = 105.384(4)°, V = 1309.23(9) Å^3, Z = 2, density$_{calcd}$ = 1.305 g cm^{-3}, T = 213 K, Cu$K\alpha$ radiation, λ = 1.54184 Å, μ = 0.722 mm^{-1}, R_{int} = 0.0308, GOF = 1.050, R_1 = 0.0459 ($I > 2\sigma(I)$), wR_2 = 0.1170 (all data), $\Delta\rho_{max,min}$ = 0.314, –0.188 eÅ^3.

Crystal data for poly-1 at 253 K: colorless needle, monoclinic, $P2_1/c$, a = 14.5783(13) Å, b = 4.8581(4) Å, c = 19.3078(12) Å, β = 105.806(8)°, V = 1315.72(19) Å^3, Z = 2, density$_{calcd}$ = 1.299 g cm^{-3}, T = 253 K, Cu$K\alpha$ radiation, λ = 1.54184 Å, μ = 0.718 mm^{-1}, R_{int} = 0.0449, GOF = 0.967, R_1 = 0.0551 ($I > 2\sigma(I)$), wR_2 = 0.1368 (all data), $\Delta\rho_{max,min}$ = 0.187, –0.219 eÅ^3.

Crystal data for poly-1 at 293 K: colorless needle, monoclinic, $P2_1/c$, a = 14.686(4) Å, b = 4.8677(11) Å, c = 19.305(4) Å, β = 106.25(2)°, V = 1324.9(5) Å^3, Z = 2, density$_{calcd}$ = 1.290 g cm^{-3}, T = 293 K, Cu$K\alpha$ radiation, λ = 1.54184 Å, μ = 0.713 mm^{-1}, R_{int} = 0.0440, GOF = 0.988, R_1 = 0.0576 ($I > 2\sigma(I)$), wR_2 = 0.1555 (all data), $\Delta\rho_{max,min}$ = 0.18, –0.31 eÅ^3.

Specific heat measurements. Thin cylindrical pellets of single-crystalline **1** and poly-**1** were prepared by compression molding. Using the pellet samples, the temperature dependence of specific heat was measured on a Quantum Design Physical Properties Measurement System (PPMS) from 2 to 300 K based on a standard relaxation method.

Thermal diffusivity measurements using a micro-temperature wave analysis (μTWA). Using thermal deposition, a sensor (serving as a thermocouple) of Au-Ni and a heater circuit of Au were prepared on SiO_2 and indium-tin-oxide (ITO) substrates, respectively. A single crystal sample of **1** or poly-**1**, whose crystallographic face was determined in advance by single-crystal X-ray analysis,

was cut to fit the size of the measuring device (~50×50×100 μm^3) and then sandwiched between the sensor and heater arranged to measure thermal diffusivity in the direction parallel and perpendicular to the polymer chains, *i.e.*, $\alpha_{M//}$, $\alpha_{P//}$, $\alpha_{M\perp}$, and $\alpha_{P\perp}$. Alternating current (AC) voltage was applied to the heater circuit by a function generator, causing periodic Joule heating at one side of the sample. The temperature change at the other side of the sample was detected as a voltage change of the thermocouple.

At a given sample temperature, the phase delay ($\Delta\theta$) of the output signal from the sensor against the input voltage to the heater circuit is represented by the following equation:

$$\Delta\theta = -\sqrt{\frac{\pi f}{\alpha}}d - \frac{\pi}{4}$$

where f is a lock-in frequency, and d is the sample length in the measurement direction. The value of α at the sample temperature can be obtained from the slope of a $\Delta\theta$–$f^{1/2}$ plot. The values of $\alpha_{M//}$, $\alpha_{P//}$, $\alpha_{M\perp}$, and $\alpha_{P\perp}$ at 300 K were measured for seven independent samples (Table S2), and the average values with confidence intervals of 95% were obtained (Figure 1e). The temperature dependence of $\alpha_{M//}$ and $\alpha_{P//}$ was measured between 11~301 K and 43~301 K, respectively (Figure 2a and Figure S1). The validation of the μTWA technique to measure thermal diffusivity has already been shown through measurements of crystalline sapphire and borosilicate glass.[S3] As shown in the above equation, if the thermal diffusivity becomes too large, the frequency of the input voltage must be increased. However, as the input frequency is increased, the intensity of the corresponding output signal becomes smaller, resulting in a measurement limitation. In the present experiments, it became difficult to accurately measure the thermal diffusivity as it approached an order of 10^{-5} m^2·s^{-1}.

3. Supporting Figure

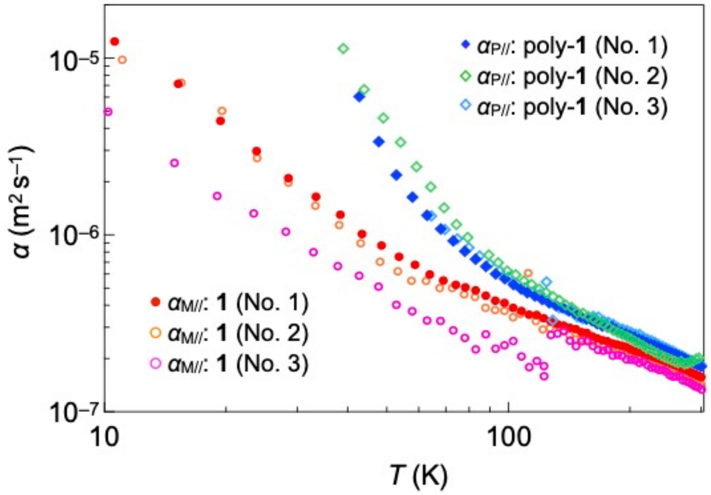

Figure S1. Temperature dependence of $\alpha_{M//}$ and $\alpha_{P//}$ for three different samples of each of **1** The data represented by red filled circles and blue filled diamonds are already shown in Figure 2a.

4. Supporting Tables

Table S1. Temperature dependence of lattice constants and unit-cell volumes for single-crystalline 1 (upper) and poly-1 (lower).

**1**	93 K	133 K	173 K	213 K	253 K	293 K
a (Å)	13.9686(5)	14.0204(5)	14.0820(7)	14.1596(6)	14.2422(8)	14.3310(6)
b (Å)	4.89373(17)	4.89521(19)	4.8982(2)	4.8989(2)	4.8999(3)	4.9168(2)
c (Å)	19.6219(6)	19.6535(8)	19.6743(10)	19.7111(7)	19.7288(10)	19.7877(8)
V (Å^3)	1318.87(8)	1325.55(9)	1333.16(11)	1342.49(10)	1350.97(13)	1367.27(11)

poly-**1**	93 K	133 K	173 K	213 K	253 K	293 K
a (Å)	14.248(3)	14.296(3)	14.3762(13)	14.4786(6)	14.5783(13)	14.686(4)
a (Å)	4.8545(7)	4.8576(7)	4.8544(3)	4.85837(17)	4.8581(4)	4.8677(11)
a (Å)	19.207(3)	19.240(3)	19.2469(12)	19.3038(7)	19.3078(12)	19.305(4)
V (Å^3)	1285.6(4)	1291.5(4)	1296.52(17)	1309.23(9)	1315.72(19)	1324.9(5)

Table S2. Values of $\alpha_{M//}$, $\alpha_{P//}$, $\alpha_{M\perp}$, and $\alpha_{P\perp}$ of seven single crystal samples of each of 1 and poly-1 at 300 K.

Sample No.	$\alpha_{M//}$ ($\times10^{-7}$ m^2 s^{-1})	$\alpha_{P//}$ ($\times10^{-7}$ m^2 s^{-1})	$\alpha_{M\perp}$ ($\times10^{-7}$ m^2 s^{-1})	$\alpha_{P\perp}$ ($\times10^{-7}$ m^2 s^{-1})
#1	1.33	1.71	0.99	0.94
#2	1.69	2.48	0.94	0.98
#3	1.85	2.25	1.31	1.32
#4	1.31	2.02	1.01	0.97
#5	1.55	2.06	1.14	1.13
#6	1.23	1.66	1.30	1.35
#7	1.31	1.87	1.42	1.48

5. Supporting References

S1. Dou, L.; Zheng, Y.; Shen, X.; Wu, G.; Fields, K.; Hsu, W. C.; Zhou, H.; Yang, Y.; Wudl, F. Single-crystal linear polymers through visible light–triggered topochemical quantitative polymerization, *Science* **2014**, *343*, 272–277.

S2. Endo, S.; Watanabe, Y.; Sasaki, T.; Fukase, T.; Toyota, N. Temperature dependence of lattice parameters of α-(BEDT-TTF)$_2$MHg(XCN)$_4$ (M=K, Rb, NH_4, and X=S, Se), *Synth. Met.* **1997**, *86*, 2013–2014.

S3. Morikawa, J.; Orie, A.; Hashimoto, T.; Juodkazis, S. Thermal and optical properties of the femtosecond-laser-structured and stress-induced birefringent regions in sapphire, *Optics Express*, **2010**, *18*, 8300.
